# Schisandrin C Improves Chronic Stress-Induced Dyslipidemia in Mice by Regulating Pyroptosis and Autophagy Levels

**DOI:** 10.4014/jmb.2501.01041

**Published:** 2025-06-23

**Authors:** Yuling Liu, Wenjun Lu, Yue Huang, Chaozhi Xu, Wanhua Wu, Zongyi Zhang, Yulin Zhang, Shanqian Li, Guixian Wu, Doudou Wang, Yao Xu, Meimei Zheng, Hongxian Wu, Lina Hu

**Affiliations:** 1 School of Public Health, Guilin Medical University, Guilin, P.R. China; 2 Key Cultivation Laboratory of Life Cycle Health Care Research, Guilin, P.R. China; 3 Guangxi Key Laboratory of Environmental Exposomics and Whole Life Cycle Health, Guilin, P.R. China; 4 General Practice Department, Affiliated Hospital of Guilin Medical University, Guilin, P.R. China; 5 Department of Pediatrics, The First Affiliated Hospital of Guangxi Medical University, Nanning, P.R. China; 6 Medical Information Management, School of Humanities and Management, Guilin Medical University, Guilin, P.R. China; 7 Communicable Disease Control Branch, Qingdao Municipal Center for Disease Control and Prevention, Qingdao, P.R. China; 8 Department of Nutrition, Second People's Hospital of Ya'an City, Ya'an City, Sichuan Province, P.R. China; 9 Department of Clinical Nutrition, Liuzhou Workers' Hospital, Liuzhou Guangxi, P.R. China; 10 Department of Cardiology, Zhongshan Hospital, Fudan University, Shanghai Institute of Cardiovascular Diseases, P.R. China

**Keywords:** Schisandrin C, chronic stress, dyslipidemia, pyroptosis, Caspase-1, autophagy

## Abstract

Chronic stress emerges as a significant risk factor for metabolic disorders, including hyperlipidemia and fatty liver disease. Schisandrin C (SchC), an essential component of Schisandra chinensis lignans, is known to possess lipid-lowering and liver-protective properties. However, the precise mechanisms underlying its action remain incompletely understood. We hypothesized that SchC exerts a protective effect on chronic stress-induced dyslipidemia in mice by modulating hepatic autophagy and pyroptosis. In this study, a dual model was established for 12 weeks, combining chronic stress with a high-fat diet (HFD). SchC or simvastatin were given respectively starting at week 10. Blood, epididymal white adipose tissue and liver were collected for further analysis. Behavioral tests and lipid levels showed that the dual model construction of HFD and chronic stress used in this study was successful in placing mice in a stressful environment and triggering dyslipidemia. SchC intervention effectively impeded the accumulation of eWAT and reduced the increased lipid levels in mice induced by chronic stress. The expression levels of the pyroptosis marker Caspase-1 and other inflammatory factors NLRP3, IL-1β, and IL-18 were significantly reduced in the liver tissues of chronically stressed mice under SchC treatment. Additionally, SchC significantly reduced expression levels of autophagy-related factors such as Beclin-1, the LC3-B/A ratio, P62, and markers related to the autophagy pathway (PI3K/AKT/mTOR). SchC effectively improves dyslipidemia through various pathways including inhibiting the PI3K/AKT/mTOR pathway, promoting autophagy, reducing pyroptosis. This provides a solid theoretical foundation for the clinical application of SchC in the treatment of dyslipidemia and related diseases.

## Introduction

In today's fast-paced society, individuals are frequently exposed to many psychophysiological stressors. Chronic stress, a non-specific adaptive response triggered by a certain level of internal and external environmental, social, and psychological factors, is a common experience [1]. When faced with increased stress levels, individuals often consume palatable foods, which helps mitigate the negative effects [2]. Chronic stress not only increases the risk of depression but also increases the likelihood of developing metabolic diseases. Prolonged chronic occupational stress can lead to elevated blood lipid and glucose levels in physicians [3]. Dyslipidemia is a risk factor for atherosclerosis [4], fatty liver disease [5], coronary heart disease [6], and other health conditions. Consequently, prolonged chronic stress can cause multisystem and multilevel damage [7].

Impairment or disruption of autophagy can contribute to a range of conditions, including obesity, nonalcoholic fatty liver disease (NAFLD), type 2 diabetes, cirrhosis, and Alzheimer's disease [8]. Our previous study has revealed that chronic stress can induce autophagy dysfunction [9]. The liver plays a crucial role in the regulation of cholesterol and triglyceride metabolism, and chronic stress can lead to significant elevation in hepatic lipid parameters, which can contribute to metabolic disorders [10]. However, after intervention with mTOR inhibitor rapamycin, lipid levels were reduced and lipid metabolism improved in mice [11]. Recent research by Wei Q and colleagues found that the activation of Caspase-1, a key regulator of pyroptosis, affects autophagic vacuole formation in HepG2 cells, suggesting that pyroptosis is closely related to autophagy [12]. Classical pathway-mediated pyroptosis involves the activation of Caspase-1, which cleaves the precursors of interleukin‐1β (IL-1β) and interleukin-18 (IL-18) and activates inflammatory factors such as IL-1β and IL-18 [13]. A clinical study found that the presence of activated forms of Caspase-1 in the liver and serum of NASH patients is strongly correlated with disease severity [14]. Chronic stress induces inflammation in liver tissues, which activates inflammatory factors, such as Caspase-1, leading to pyroptosis and contributing to the progression of NAFLD [15].

Dyslipidemia is a prevalent global issue that is affecting individuals at an increasingly younger age [16]. Schisandra chinensis (SC) has been used clinically to treat hepatitis and cirrhosis because of its hepatoprotective function [17]. Schizandrin A, Schizandrin B, Gomisin G, Schisandrin C (SchC) are more prevalent in SC, and the majority of research on SC lignans has concentrated on the first two monomers [18]. Recently, it has been discovered that SchC can be used at relatively low doses to produce better outcomes. For instance, Han J *et al*. observed that SchC was helpful in alleviating vascular endothelial dysfunction in mice at lower dose [19]. In addition, SchC has a number of biological effects, including promoting autophagy [20], preventing pyroptosis [21], reducing inflammation [22], and reducing oxidative stress [23]. According to recent research, SchC reduces inflammation and lipid metabolism to help people with hepatic fibrosis [24]. SchC inhibits lipid accumulation by regulating adipogenesis and lipolysis in 3T3- L1 adipocytes [25]. However, the role of SchC in dyslipidemia induced by chronic stress combined with a high-fat diet (HFD) and its mechanisms are unknown.

We hypothesized that SchC exerts a protective effect on chronic stress-induced dyslipidemia in mice by modulating hepatic autophagy and pyroptosis. In order to simulate the chronic stress environment and unhealthy eating habits of contemporary people, the animal model adopted the double model of chronic stress combined with HFD used in our previous study to explore the effect of SchC on dyslipidemia induced by chronic stress combined with HFD by weighing visceral fat and measuring blood lipid level. To determine the underlying mechanism, we also evaluated the expression of pyroptosis markers, autophagy-related factors, as well as inflammatory cytokines in the liver.

## Materials and Methods

### Drugs

**Fig. 1:**
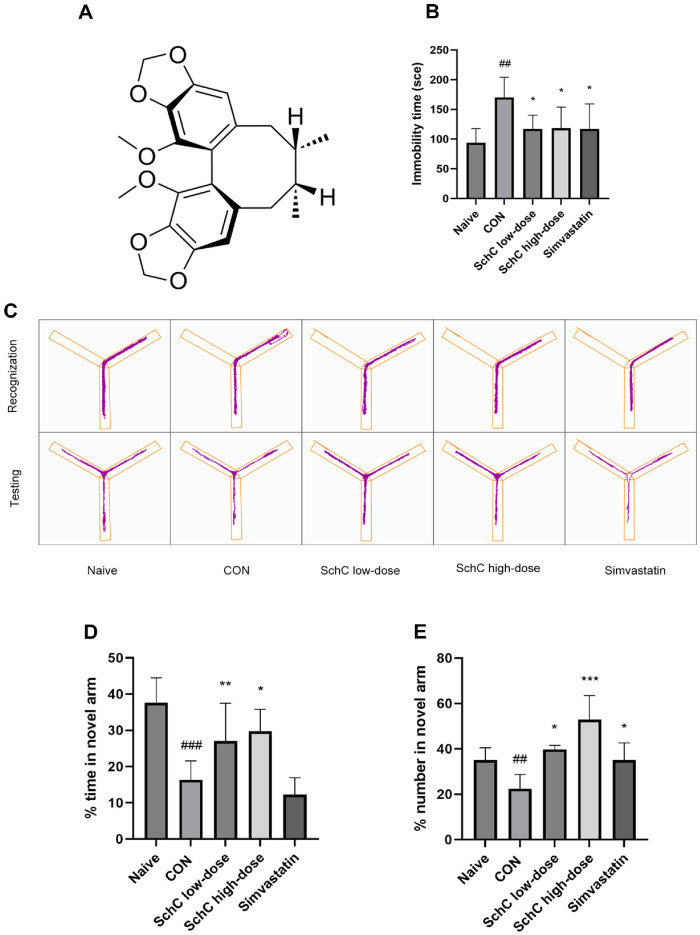
Schisandrin C improves chronic stress-induced depressive-like behavior. (**A**) Structure of Schisandrin C; (**B**) Forced swim test immobilization time; (**C**) Y-maze experiment new arm identification and test trajectory diagram; Y-maze experiment (**D**) ratio of the number of entries into the new arm, and (**E**) ratio of the dwell time. Data are expressed as mean ± SD (*n* = 6-10). ##*p* < 0.01 and ###*p* < 0.001, vs. Naive group. *p < 0.05, **p < 0.01 and ***p < 0.001, vs. control group. Naive: mice receiving normal chow and no chronic stress, CON: HFD + chronic stress, SchC low-dose: CON + 2.5 mg/kg SchC, SchC highdose: CON + 5 mg/kg SchC, and Simvastatin: CON + 20 mg/kg Simvastatin. High-fat diet and chronic stress were continued for 12 weeks, with the appropriate pharmacological intervention given at week 10.

Schisandrin C (SchC) (Fig. 1A) purchased from Source Ye Company (batch No.: 61301-33-5). Simvastatin, purchased from Hangzhou MSD Pharmaceutical Co., Ltd., USA (batch No.: J20180007). high-fat diet (HFD)(78.85%ND, 21% lard oil, 0.15%cholesterol; Beijing Botai Hongda Biotechnology Co., Ltd., China).

### Animals

Sixty Male C57BL/6J mice, aged between 7 to 11 weeks and weighing 15 to 30 grams, were carefully selected and purchased from Hunan Slake Jinda Laboratory Animal Co. Ltd., China (SCXK Xiang: 2019-0004). These animals were housed in the SPF laboratory of Guilin Medical College, where a 12-h day-night cycle was maintained with indoor temperatures ranging between 20 and 24°C and the humidity levels were maintained between 40% and 60%. The animals had access to food and water and were allowed to acclimate for a week prior to conducting the experiments. The Laboratory Animal Ethics Committee of Guilin Medical College (GLMC20243268) has approved all animal experiments, which were carried out in accordance with relevant national laws and ethical requirements for animal testing.

### Grouping of Experimental Animals

Five experimental groups were established as follows: Naive group [mice receiving standard chow and no chronic stress, n=10], Control group (CON) [mice receiving HFD + chronic stress + physiological saline, n=15], SchC low-dose group [CON + 2.5 mg/kg SchC, n=15], SchC high-dose group [CON + 5 mg/kg SchC, n=15] and Simvastatin group [CON + 20 mg/kg Simvastatin, n=15].

### Dual Modeling of Persistent Stress in Combination with an HFD

In this experiment, chronic stress combined with the HFD dual model was used, and the modeling process lasted 12 weeks [26]. After a 1-week acclimatization period, all mice in the Naive group were fed a regular diet, while the rest of the mice were fed a HFD and chronic stress and were randomly exposed to two different stimuli at a fixed time each day for the entire experiment. The Naive group mice were allowed to move freely without receiving any stimuli. The stimulation types included 50 ml centrifuge tube binding (both horizontally and vertically), a wet pad, pad removal, cage tilt at 45°C, overnight illumination, and tail clip. It was ensured that the same stimulus did not occur consecutively every day, as detailed in Table 1 [27].

### Dosage

Starting from week 10, each group received the corresponding intervention drugs based on their daily exposure to continuous stress and high-fat diet (HFD). The specific details are as follows: (1) The model group received 10%DMSO by gavage, (2) the SchC low-dose group received a 2.5 mg/kg SchC solution by gavage, (3) the SchC high-dose group received a 5 mg/kg SchC solution by gavage, and (4) the Simvastatin group received a 20 mg/kg dose of simvastatin by gavage. The daily gavage dose was determined based on a combination of literature and the body weight of the mice on that particular day [28, 30]. The pharmacological intervention lasted for a period of 3 weeks.

### Sample Collections

Mice were anesthetized with sodium pentobarbital (50 mg/kg) and underwent cardiac blood extraction followed by saline perfusion. After a blood sample was collected, it was left on ice for 30 min. Subsequently, the sample was centrifuged in a high-speed freezer at 4°C for 5 min at 3,500 rpm to separate the upper serum. The resulting serum was then stored in a -20°C refrigerator for future use. The adipose tissue from both the liver and the epididymal white adipose tissue (eWAT) was carefully collected, quickly frozen in liquid nitrogen, and finally stored at -80°C in a reserve refrigerator.

### Behavioral Tests

**Y-maze.** The Y-maze comprises three identically shaped and sized arms that are angled 120 degrees apart. Each arm has a freely movable spacer groove in the middle that keeps it apart from the others. After installing the spacer in one arm, the mice were positioned in the same way in the unspacerized arm and allowed to roam unhindered for ten min. Once an hour had passed, the spacer of the novel arm was removed, and the mice were positioned in the same spot within the Y-maze. They explored for five minutes, during which time the number of times they entered the novel arm was noted. The Y-maze was wiped with alcohol after each mouse was trained or tested to eliminate the influence of residual odor on the experimental results [31].

### Forced Swim Test (FST)

The mice were placed individually in a 20 cm high, 14 cm diameter organocircular glass tank that held 16 cm of water. The water temperature was kept at 25 ± 1°C, while the room temperature was kept at 23°C. The total time of each forced swim test was 6 min, with 2 min of adaptation to swimming and 4 min of immobility time. Immobility time was defined as the time when the mouse floated motionless on the water surface with only a slight swing of its tail and forepaws to maintain body balance and keep its head above water [32].

### Biochemical Marker Analysis

Blood was obtained from mice through cardiac blood sampling, followed by centrifugation at 4°C for 30 min after standing on ice. The upper layer of the serum was carefully collected and the levels of TG, LDL-c, and HDL-c in the serum were analyzed using a biochemical kit, following the instructions provided. For the markers of pyroptosis Caspase-1 and IL-1β in the serum of mice were measured by ELISA.

### Western Blot (WB) Analysis

Liver tissues were lysed in the RIPA lysis buffer containing 1% phosphatase inhibitor cocktail and 1% protease inhibitor cocktail (RPP) on ice and centrifuged (12,000 g, 4°C and 30 min). The protein concentration was accurately measured using a BCA assay kit. The uptake volume (30-50 μg/lane) was calculated to ensure consistency for each sample. The hierarchical separation of proteins from all samples was achieved using 7.5-12%SDS gels, followed by wet transfer of the proteins onto PVDF membranes (0.2 μm pore size membrane for small molecular weight proteins and 0.45 μm pore size membrane for large molecular weight proteins). The membrane was transferred for 90-120 min, followed by incubation with 3% BSA or 3% skimmed milk for 1 h, washing with TBST for 15 min, and placing the membrane in a configured primary antibody. The membrane was then incubated overnight at 4°C with the primary antibody. After washing with TBST for 15 min the next day, the cells were incubated with horseradish peroxidase (HRP)-conjugated secondary antibodies (dilution of 1:10,000) for 1 h at room temperature. At the end of the secondary antibody incubation, the cells were washed with TBST six times for 10 min each time and developed using an ultrasensitive chemiluminescence kit and a hemiluminescence fully automated imaging system was used for the development. Antibody details are presented in Table 2.

### Immunohistochemistry Assay

The liver tissues were carefully fixed in 4% paraformaldehyde (20 times the volume of the liver) for 2 d. Then, the liver tissue was embedded in paraffin and the sections were prepared. Sections were routinely dewaxed in water and antigen-repaired 5% Bovine serum albumin (BSA) blocking solution was added and the sections were incubated at 37°C for 30 min. The sections were incubated with LC3, P62 and Caspase-1 antibodies at 4°C overnight and then incubated with the secondary antibodies. DAB (diaminobenzidine tetrahydrochloride) developer solution was used for color rendering, and Mayer’s hematoxylin was added for redyeing. Neutral gum sealing was performed, and three fields in each group were randomly selected under a 400 × light microscope for image acquisition. The presence of brownish-yellow particles on the tissue is a positive area. Image Proplus was used to calculate the average optical density of the positive region of immunohistochemical tissues.

### Statistical Analysis

All the statistical analysis operations of the data in this experiment were performed with GraphPad Prism 10 software. Data are presented as mean ± standard deviation (SD). Statistical differences were compared using one-way ANOVA followed by Dunnett's post hoc test or *t*-test. Differences were considered significant at *p* < 0.05.

## Results

### Schisandrin C Improves Chronic Stress-Induced Depressive-Like Behavior

After the 12-week experiment was over, Y-maze and forced swim test (FST) studies were carried out in the same setting to determine whether chronic stress generates a stressful environment for mice. In comparison to the naive group, mice who experienced chronic stress remained in the water tank for a longer period of time (*P* < 0.01), according to the results of FST trials (Fig. 1B). Y-maze testing showed that chronically stressed mice exhibited a statistically significant reduction in novel arm entries (*P* < 0.01) and residence time (*P* < 0.001) compared to the naïve group (Fig. 1C-1E). Thus, the current study concluded that micés mood and memory were negatively impacted by prolonged stress. However, the FST experiment results showed a significant decrease in the duration of immobilization in the FST (*P* < 0.01, *P* < 0.05) following the administration of Schisandrin C (SchC) to the mice. The Y-maze data indicated increased exploration of the novel arm, with longer stay duration (*P* < 0.01, *P* < 0.05) and more frequent entries (*P* < 0.05, *P* < 0.001). SchC can thereby improve mood issues and memory. SchC demonstrated a more pronounced improvement in memory at high doses, while its effect on mood was more significant at low doses. After simvastatin intervention, only a reduction in immobility time in the FST (*P* < 0.05) and an increase in the number of entries into the novel arm in the Y-maze test (*P* < 0.05) were observed in mice. Thus, the current investigation found that SchC was superior to simvastatin in reducing depression-like symptoms brought on by chronic stress. In summary, the chronic stress model employed in this study created a stress-inducing environment that adversely affected both memory and mood in mice, while SchC demonstrated significant alleviating effects.

### Effect of Schisandrin C on Body Weight and the eWAT in Chronic Stress-Induced Dyslipidemic Mice

**Fig. 2:**
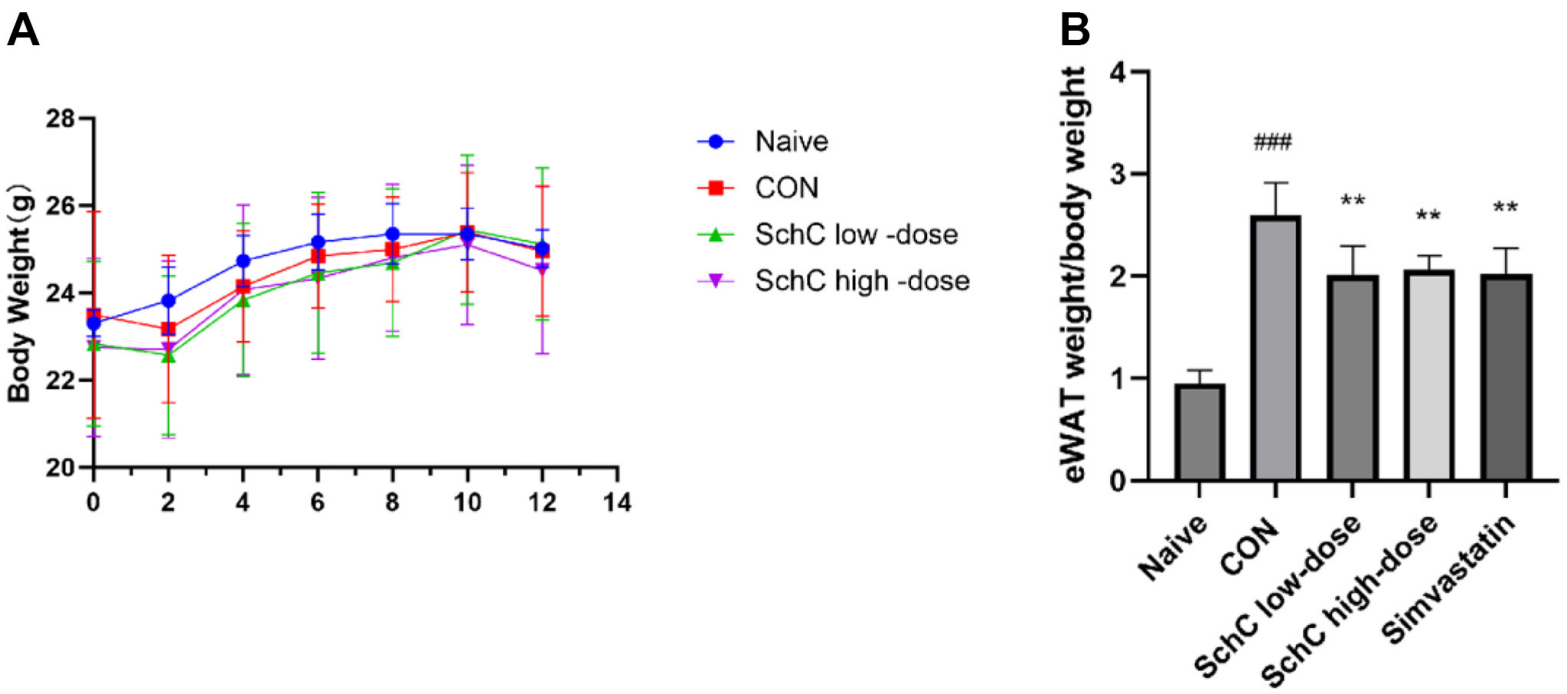
Effects of Schisandrin C on Body Weight and Epididymal white adipose tissue weight in Mice. (**A**) No significant differences in body weight were observed among the mice; (**B**) Epididymal white adipose tissue weight to body weight. Data are expressed as mean ± SD (*n* = 8-10). ###*p* < 0.001, vs. Naive group. **p < 0.01, vs. control group. Naive: mice receiving normal chow and no chronic stress, CON: HFD + chronic stress, SchC low-dose: CON + 2.5 mg/kg SchC, SchC highdose: CON + 5 mg/kg SchC, and Simvastatin: CON + 20 mg/kg Simvastatin. High-fat diet and chronic stress were continued for 12 weeks, with the appropriate pharmacological intervention given at week 10.

Over a 12-week period, we tracked the micés weight fluctuations consistently. Mice on a high-fat diet (HFD) typically gain a considerable amount of weight[33]. Nonetheless, the study's findings demonstrated that the Naive and CON groups saw weight increase at comparable rates. This suggests that chronic stress decreased the rate at which mice gained weight as a result of an HFD. Meanwhile, SchC had no significant effect on the body weight of mice (Fig. 2A). The weight gain induced by a HFD is typically accompanied by the accumulation of epididymal white adipose tissue (eWAT) in the body. In this work, we discovered that the mice in the CON group had considerably more weight in their eWAT than the mice in the naive group (*p* < 0.001). This suggests that a combination of chronic stress and a high-fat diet (HFD) led to the deposition of significant amounts of visceral fat. However, following SchC intervention, mice in the SchC low-dose (*p* < 0.01) and SchC high-dose (*p* < 0.01) groups had significantly lower eWAT weights than those in the CON group; the impact was more pronounced in the SchC high-dose group (Fig. 2B). Following simvastatin treatment, micés eWAT weight also decreased significantly (*p* < 0.01). Simvastatin’s effect on visceral fat reduction was similar to that of the SchC high-dose group. This suggests that while the adverse effects of chronic stress are clinically subtle yet detrimental, SchC effectively mitigates visceral adipose accumulation induced by both chronic stress and HFD, proposing a novel therapeutic strategy for related metabolic disorders.

### Schisandrin C Modulates Lipid Levels in Chronic Stress-Induced Dyslipidemic Mice

**Fig. 3:**
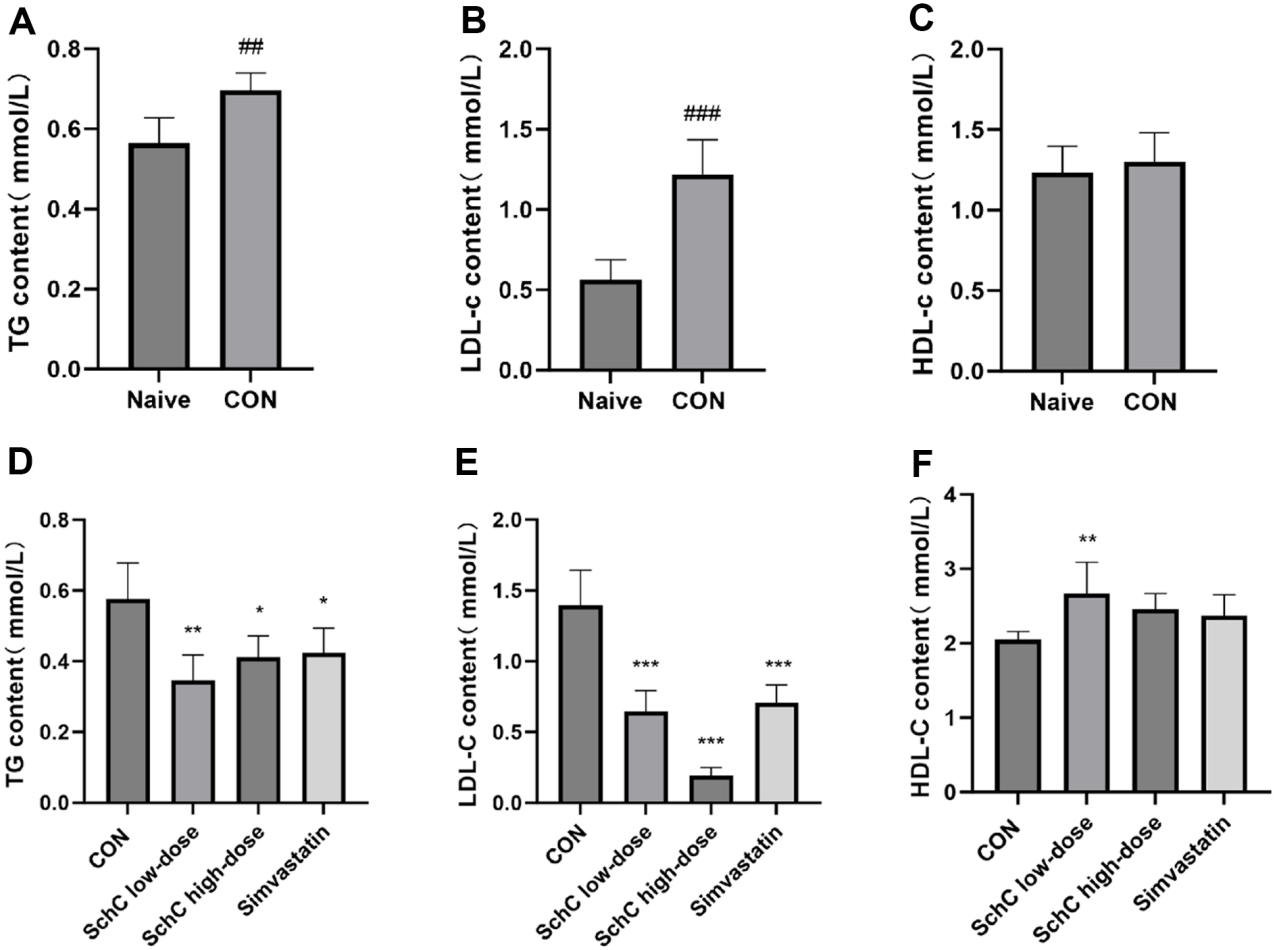
Schisandrin C modulates lipid levels in chronic stress-induced dyslipidemic mice. (**A**) and (**D**) TG; (**B**) and (**E**) LDL-c; (**C**) and (**F**) HDL-c. Data are expressed as mean ± SD (*n* = 8-10). ##*p* < 0.01 and ###*p* < 0.001, vs. Naive group. *p < 0.05, **p < 0.01 and ***p < 0.001, vs. control group. Naive: mice receiving normal chow and no chronic stress, CON: HFD + chronic stress, SchC low-dose: CON + 2.5 mg/kg SchC, SchC high-dose: CON + 5 mg/kg SchC, and Simvastatin: CON + 20 mg/ kg Simvastatin. High-fat diet and chronic stress were continued for 12 weeks, with the appropriate pharmacological intervention given at week 10.

A major risk factor for several illnesses, such as atherosclerosis, cardiovascular disease, and nonalcoholic fatty liver disease (NAFLD), dyslipidemia is a major global public health concern. According to case-control research, coal miners have a greater incidence of dyslipidemia and are more vulnerable to occupational stress due to their unique work environment than the general population [34]. Our previous study demonstrated that chronic stress can lead to elevated blood lipid levels in mice [9]. We first investigated whether HFD combined with chronic stress would lead to dyslipidemia in mice. The results showed that compared to the naive group, the TG (*p* < 0.01) and LDL-c (*p* < 0.001) in the CON group were significantly higher, but the HDL-c (p > 0.05) did not change significantly (Fig. 3A-3C). We concluded that the dual model of HFD and chronic stress led to dyslipidemia in mice. Following that, we looked at the micés blood lipid levels to see if SchC controls the aberrant alterations in serum lipid biochemical indices brought on by chronic stress and HFD. When compared to the CON group, the administration of either simvastatin (*p* < 0.05) or SchC (SchC low-dose group *p* < 0.01; SchC high-dose group *p* < 0.05) significantly decreased serum TG levels. Serum LDL-c levels were considerably lowered by the administration of either SchC (SchC low-dose group *p* < 0.001; SchC high-dose *p* < 0.001) or simvastatin (*p* < 0.001), and the improvement impact of SchC was significantly greater than that of simvastatin in a dose-dependent manner. Furthermore, serum HDL-c levels were markedly raised by the SchC (SchC low-dose group *p* < 0.01) intervention (Fig. 3D-3F). SchC may therefore help with dyslipidemia brought on by chronic stress.

### Schisandrin C Reverses Chronic Stress-Induced Autophagy Damage in Liver Tissue of Dyslipidemic Mice

**Fig. 4:**
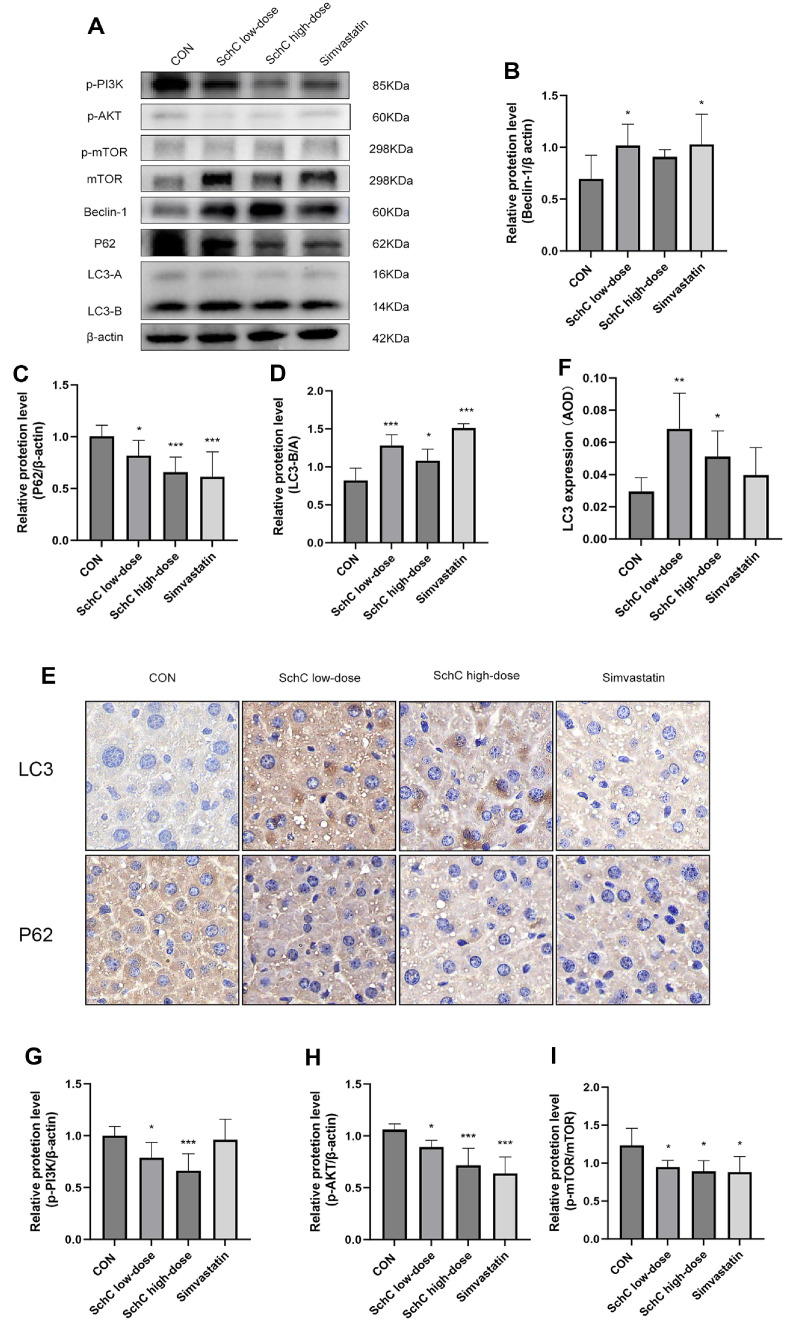
Schisandrin C reverses chronic stress-induced autophagy damage in liver tissue of dyslipidemic mice. (**A**) The activation of Phospho-PI3K (p-PI3K), Phospho-AKT (p-AKT), Phospho-mTOR (p-mTOR), Beclin-1, P62 and LC3 in liver of mice was analyzed using western blotting. Quantitative analysis of (**B**) Beclin-1, (**C**) P62, (**D**) LC3, (**E**) p-PI3K, (**F**) p-AKT, and (**G**) p-mTOR was performed using ImageJ software. (**H**) Positive staining of LC3 and P62 in liver tissues was detected by IHC staining. 400x. (i) LC3 quantitative analysis. Data are expressed as mean ± SD (*n* = 3 or 6). *p < 0.05, **p < 0.01 and ***p < 0.001, vs. control group. CON: HFD + chronic stress, SchC low-dose: CON + 2.5 mg/kg SchC, SchC high-dose: CON + 5 mg/kg SchC, and Simvastatin: CON + 20 mg/kg Simvastatin. High-fat diet and chronic stress were continued for 12 weeks, with the appropriate pharmacological intervention given at week 10.

Hepatic lipid deposition may worsen if intracellular lipid buildup in the liver interferes with autophagy [35]. Numerous studies have confirmed that chronic stress triggers autophagy defects [36, 37]. Western Blot (WB) was utilized to identify autophagy-related components and pathway protein expression in mouse liver tissues in order to examine if SchC might improve autophagy abnormalities brought on by chronic stress (Fig. 4A). The findings indicated that the initiation of autophagy was improved because the expression of Beclin-1 protein was considerably higher in the SchC low-dose group following SchC administration than in the CON group (*p* < 0.05)(Fig. 4B). Both the SchC high-dose group (*p* < 0.05) and the SchC low-dose group (*p* < 0.001) showed a significant decrease in P62 protein levels (Fig. 4C). Both the SchC low-dose group (*p* < 0.001) and the SchC high-dose group (*p* < 0.05) showed a substantial rise in the LC3-B/A ratio, indicating a greater production of autophagosomes. The effect of the low dose was more noticeable (Fig. 4D). Immunohistochemistry results demonstrated that following SchC intervention, LC3 expression increased dramatically and that the difference was statistically significant. Following SchC administration, there was a significant decrease in P62 expression (Fig. 4E and 4F). The change trend was also consistent with WB data. Pathway proteins showed a statistically significant decrease in levels of Phospho-PI3K (p-PI3K), Phospho-AKT (p-AKT), and Phospho-mTOR (p-mTOR) in both the SchC low and high dose groups (Fig. 4G-4I). Autophagy was also activated by simvastatin, although SchC was more effective in increasing Beclin-1 and LC3 expression and decreasing p-mTOR and P62 protein levels. In conclusion, by blocking the PI3K/AKT/mTOR pathway, SchC successfully repaired the autophagy damage in the mouse liver, opening up a new therapeutic avenue for the management of associated liver disorders.

### Schisandrin C Improve Chronic Stress-Induced Dyslipidemia by Reducing Pyroptosis

**Fig. 5:**
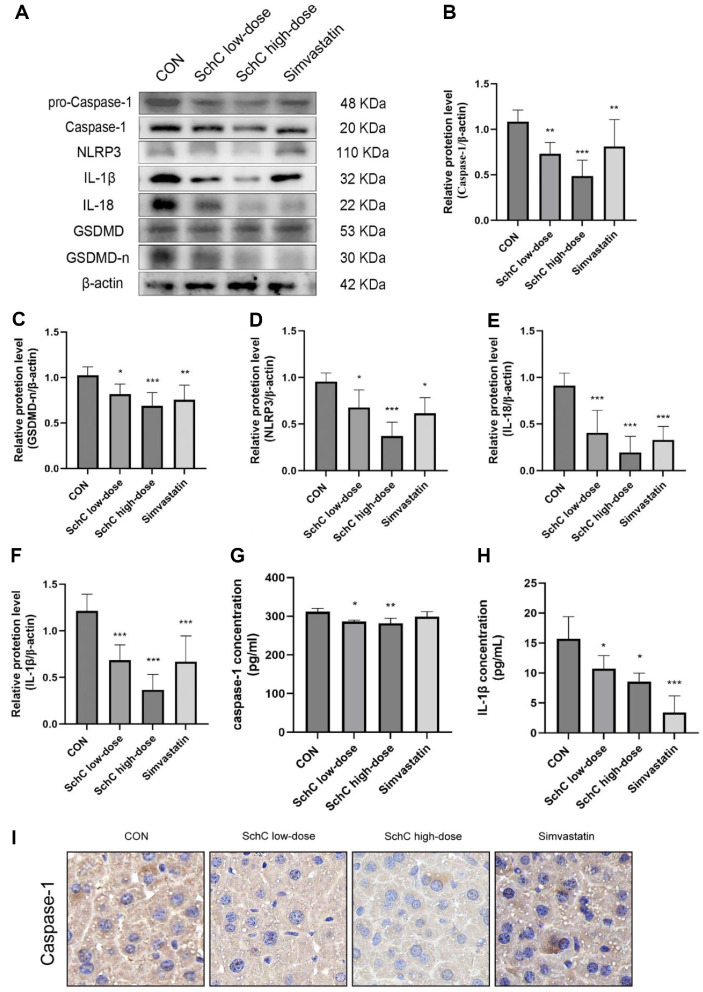
Schisandrin C Improve chronic stress-induced dyslipidemia by reducing pyroptosis. (**A**) The pro-Caspase-1, Caspase-1, NLRP3, IL-1β, IL-18, Full length GSDMD (**GSDMD**) and cleaved N-terminal GSDMD (GSDMD-n) in liver of mice was analyzed using western blot. Quantitative analysis of (**B**) Caspase-1, (**C**) GSDMD-n, (**D**) NLRP3, (**E**) IL-1β and (**F**) IL-18 was performed using ImageJ software. The (**G**) Caspase-1 and (**H**) IL-1β level of serum was assessed by ELISA. (**I**) Positive staining of Caspase-1 in liver tissues was detected by IHC staining. 400x. Data are expressed as mean ± SD (*n* = 3 or 6). *p < 0.05, **p < 0.01 and ***p < 0.001, vs. control group. CON: HFD + chronic stress, SchC low-dose: CON + 2.5 mg/kg SchC, SchC high-dose: CON + 5 mg/kg SchC, and Simvastatin: CON + 20 mg/kg Simvastatin. High-fat diet and chronic stress were continued for 12 weeks, with the appropriate pharmacological intervention given at week 10.

Previous studies have revealed that chronic stress can lead to pyroptosis [15]. Using WB, we identified pyroptosis-related proteins in mouse liver tissues and investigated whether SchC had an anti-cell pyroptosis effect on chronic stress-induced dyslipidemia in mice. Pyroptosis is initiated by Caspase-1 activation, and cell rupture death is indicated by the production of cleaved N-terminal GSDMD (GSDMD-n). Results showed that compared with the CON group, both SchC and simvastatin treatment significantly reduced the expression of Caspase-1 and GSDMD-n (*p* < 0.05) (Fig. 5B and 5C). Moreover, the expression levels of other pro-inflammatory factors—NLRP3 (*p* < 0.05), IL-1β (*p* < 0.001), and IL-18 (*p* < 0.001)—were also markedly decreased (Fig. 5D-5F). The reduction in NLRP3, IL-1β, and IL-18 expression indicated an attenuation of inflammatory response. Notably, as the SchC dosage increased, the downregulation of pyroptosis-related proteins became more pronounced. However, the inhibitory effect of simvastatin on pyroptosis was less significant than that of the high-dose SchC group. We used ELISA to assess the amounts of Caspase-1 and IL-1β in the serum of mice after SchC gavage in order to ascertain whether SchC regulates pyroptosis. Compared with the CON group, SchC effectively suppressed the expression of Caspase-1 (*p* < 0.05, *p* < 0.01) and IL-1β (*p* < 0.05, *p* < 0.05) in mice serum (Fig. 5G and 5H). Following the intervention of SchC, immunohistochemical tests revealed that the liver's expression of Caspase-1 was suppressed (Fig. 5I). The change trend was also consistent with WB data. The difference was statistically significant, and the pattern was in line with the WB findings. Consequently, SchC has demonstrated efficacy in mitigating chronic stress-induced pyroptosis, suggesting a novel therapeutic strategy for liver disorder management.

## Discussion

Dyslipidemia has emerged as a major global public health challenge [38]. In the United States, statin use among adults increased by 79% between 2002 and 2013 [39]. With rapid economic growth, lifestyle changes, and unhealthy habits, China has nearly doubled the age-standardized prevalence of hypercholesterolemia, increasing from 4.9% to 8.2% between 2015 and 2018 [40]. While the prevalence of dyslipidemia in China is lower than that in the United States (42.7% and 56.8%, respectively), the rates of awareness, treatment, and control of dyslipidemia in the U.S. are three, four, and seven times higher than those in China, respectively [41].

Statins are a class of drugs commonly used to treat hyperlipidemia by inhibiting the synthesis and increasing LDL receptors in the liver [42, 43]. Simvastatin, a highly lipophilic statin, crosses the blood-brain barrier and alleviates chronic stress-induced depressive symptoms while treating dyslipidemia [44]. Therefore, this study used simvastatin as a positive control group to compare the efficacy of Schisandrin C (SchC) with simvastatin. Simvastatin also has the potential for hepatotoxicity, as evidenced by increased levels of alanine aminotransferase, aspartate aminotransferase, and serum total bilirubin [45, 46]. The widespread and potentially unsafe use of statins underscores the importance of developing safer and more effective medications to combat dyslipidemia that is induced by chronic stress and a high-fat diet (HFD). Schisandra chinensis (SC), an herbal remedy with a low toxicity profile and minimal side effects, has been used for over 2,000 years [47]. Studies on hyperlipidemic mice fed a HFD showed that the methanolic extract of Schisandra Fructus lowered total cholesterol and triglyceride levels and inhibited lipid peroxidation in hepatic tissue without altering body weight [48]. *In vitro* studies demonstrated that SC inhibited adipogenesis and ameliorated TG accumulation by downregulating the expression of PPARγ, C/EBPα, and FAS adipocyte-specific transcription factors in 3T3-L1 cells [49]. From the behavioral data, consistent with the previous results [9], the chronic stress procedure used in this experiment has created a stressful environment in mice similar to that of humans. SchC has a mitigating effect on anxiety-depression-like behavior in mice. The serum TG and LDL-c of control mice were significantly higher than those of the naive group, suggesting that the dual-model structure of HFD and chronic stress resulted in dyslipidemia in mice. SchC reduced plasma TG and LDL-C levels while increasing HDL-C, and concurrently decreased eWAT mass in mice with dyslipidemia induced by chronic stress plus HFD feeding. The reason why SchC reduces LDL-c more than TG may be related to the differences in the pathways of TG and LDL-c metabolism and the specificity of the target of action of SchC. LDL-c is cleared mainly by endocytosis mediated by LDL receptors on the surface of hepatocytes, a process that is regulated by precise regulation, *e.g.*, by the PCSK9-regulated LDL receptor [50]. The synthesis of LDL-c is single-regulated, and cholesterol is affected by the enzyme HMG-CoA reductase, and direct inhibition of this enzyme reduces cholesterol production [51]. The majority of the complex metabolic route of TG is made up of endogenous and exogenous metabolic pathways, which are controlled by several variables. Additionally, the TG level is strongly correlated with insulin resistance, nutrition, and other factors [52]. Simvastatin decreases cholesterol synthesis by blocking HMG-CoA reductase and up-regulates LDL receptor expression, which greatly reduces LDL-c [53]. Experimental results demonstrate that SchC's effects on lowering TG and LDL-c are comparable to simvastatin. We speculate that SchC's greater efficacy in reducing LDL-c versus TG may involve the same target as simvastatin but with enhanced activity, suggesting multi-mechanistic LDL-c lowering effects that require further investigation.

The Caspase-1-mediated pyroptosis pathway is an active mechanism of cell death that maintains the stability of the internal environment. Our previous study found that increased Caspase-1 expression was observed in multiple tissues of mice with chronic stress [54]. Caspase-1 knockout in chronically stressed mice reversed depression-like behavior caused by chronic stress, and it also suppressed IL-1β expression, a downstream inflammatory factor of Caspase-1 [55]. Some studies have found that Caspase-1 is associated with steatosis, and treatment with the Caspase-1 inhibitor VX765 in mice with alcohol-induced liver injury results in reduced hepatic lipid deposition and lower serum TG levels, which in turn decreases liver injury [56]. Based on these findings, we conclude that Caspase-1 is a potential target for treating dyslipidemia by regulating pyroptosis. The results of several experiments showed that the results of the present study showed reduced hepatic Caspase-1 expression in chronic stress-induced dyslipidemic mice under SchC treatment as well as WB results showed inhibition of GSDMD-n expression inhibited pyroptosis. Additionally, there is evidence that Schisandrin B reduces Colonic tissue pro-Caspase-1 and expression and Gasdermin D protein levels by regulating pyroptosis, which in turn alleviates the loss of epithelial cells in colitis [57].

Chronic stress over an extended period can result in elevated systemic inflammation [54]. Our previous research demonstrated that persistent chronic stress leads to the expression of NLRP3 inflammatory vesicles, IL-1β, and IL-18 in mouse brain tissue [9]. Wang *et al*.’s research revealed that naringenin hindered the onset of nonalcoholic fatty liver disease (NAFLD) by suppressing NLRP3 inflammasome activation, preventing Caspase-1 lysis, and mitigating liver inflammation in mice [58]. MCC950 (NLRP3 inflammasome inhibitor) suppresses the expression of NLRP3 and Caspase-1 p10 and inhibits lipid accumulation by down-regulating SREBP 1 and SREBP 2 [59]. The suppression of the Caspase-1 gene was capable of preventing the increase in the mRNA and protein levels of the downstream target IL-1β, as a result of chronic stress [55]. Nevertheless, IL-1β promoted neutral lipid accumulation in lung mesenchymal cells [60]. The role of inflammatory factors in lipid metabolism is widely recognized. Our study found that SchC reduced the protein expression levels of NLRP3, IL-1β, and IL-18 in the liver of chronic stress-induced dyslipidemic mice. Among all SC lignan extracts tested, SchC demonstrated the highest efficacy in suppressing both NLRP3 inflammasome activation and IL-1β secretion [21]. These findings suggest that SchC ameliorates chronic stress-induced dyslipidemia by modulating pyroptosis and inflammation.

Inhibition of autophagy and significant reduction of HDL-c were observed in dyslipidemia, atherosclerosis, and non-alcoholic fatty liver disease [61, 62]. The 3-MA autophagy inhibitor was found to significantly decrease intercellular HDL-c in in vitro tests. Furthermore, the amount of intercellular HDL-c was suppressed when small interfering RNA down-regulated the autophagy gene Atg 7 in hepatocytes [63]. When hepatocytes were treated with the Autophagy activator rapamycin, HDL-c secretion was significantly increased [64]. Autophagy activator rapamycin treatment to diabetic mice has been shown to significantly raise blood HDL-c levels in animal tests [65]. Furthermore, HDL-c levels can rise when autophagy is activated through medication and other methods. Thus, there is a strong correlation between autophagy levels and HDL-c levels. Autophagosomes transport degraded contents to lysosomes for degradation, and LC3B is involved in the formation of autophagosomes, so LC3-B/A protein levels are used as an accepted indicator for the evaluation and determination of autophagy [66]. Upon encapsulation of P62 into autophagosomes, they undergo degradation by proteolytic enzymes within autolysosomes. When autophagy is impaired, P62 cannot be degraded by these enzymes, leading to its accumulation within cells and the formation of ubiquitin-positive inclusion bodies. Thus, P62 is a marker of impaired autophagy function [67]. Our preliminary study found that the mRNA expression levels of Beclin-1 and LC3-B/A were reduced, while those of P62 and mTOR were increased in the whole brain tissues of depressed mice stimulated by chronic stress [9]. In addition, clinical studies have revealed that autophagy is low in the blood of depressed patients [68]. When autophagy is activated in hepatocytes, it selectively recognizes excess lipid droplets in the liver and fuses them with lysosomes, where the triglycerides are hydrolyzed, thereby removing excess fat from the liver [69, 70]. Thus, activating autophagy can ameliorate dyslipidemia from multiple pathways [71]. We utilized the Western Blot (WB) method to evaluate the expression levels of total autophagy-related proteins in the liver of mice under chronic stress and discovered that SchC reversed the expression of beclin-1, P62, and LC3-B/A-autophagy-related proteins in the liver of mice with chronic stress-induced dyslipidemia, which concurred with Yan L *et al*. findings [72]. At low SchC dosages, autophagy-associated protein activity is increased. According to Choi H, the study's findings indicate that SC can exhibit autophagy suppression during more extensive metered dosing [73]. Furthermore, at modest dosages, medications have less harmful effects on mice. In general, schisandra is not overly high when taken as a dietary supplement. As a result, it is highly valuable as a nutritional treatment and as a pharmacological monomer. In conclusion, SchC reduces hepatic lipid deposition caused by chronic stress-induced dyslipidemia by activating autophagy. Chatterjee *et al*. found that Caspase-1 inhibitor VX765 inhibited caspase 1-mediated IL-1β production and Gasdermin D processing, enhanced LC3 expression in lesions, and was effective in alleviating vascular inflammation and atherosclerosis [74]. Additionally, Caspase-1 inhibitor Z-YVAD-FMK significantly blocked autophagic cell death, which was characterized by the downregulation of Beclin-1 and LC3B [75]. This suggests that Caspase-1 and autophagy have a mutual regulatory mechanism.

mTOR is a crucial regulator of autophagy, controlling nucleation, autophagosome elongation, autophagosome maturation, and termination, and participates in the negative regulation of autophagy [76]. PPDPF has been reported to inhibit hepatic steatosis by suppressing mTORC1 activity by disrupting the Raptor-DDB1 interaction [77]. High levels of atmospheric ammonia regulate lipid metabolism in porcine skeletal muscle by increasing lipid synthesis and decreasing β-oxidation by activating mTOR signaling and inhibiting AMPK signaling [78]. Moreover, mTORC1 can promote lipogenesis by upregulating SREBP1, which is downstream of mTORC1 [79]. Therefore, mTOR is an important target for the regulation of hepatic lipid homeostasis [80]. However, chronic stress induces mTOR-dependent inhibition of autophagy, leading to cell death [81]. Previous studies have demonstrated that SC reduces lipopolysaccharide-induced mammary gland injury by inhibiting suppression of the mTOR signaling pathway [82]. Therefore, we examined the expression of mTOR and related pathway proteins using WB. The results showed that SchC decreased the expression levels of p-PI3K, p-Akt, and p-mTOR in the livers of chronic stress-induced dyslipidemic mice, indicating that SchC restored chronic stress-induced dyslipidemia in mice by inhibiting the PI3K/Akt/mTOR signaling pathway of autophagy injury.

The results demonstrated that the intervention impact of this experiment was comparable to that of the higher stoichiometry of SC described in the literature, even though the SchC stoichiometry employed in this experiment was lower than the intervention stoichiometry of SC reported in the literature. SchC is therefore more consistent with the idea of seeking safety and effectiveness in contemporary medicine because it is low in toxicity and has no adverse effects. It is anticipated to become a more viable therapeutic drug choice and pave the way for the treatment of linked disorders. It offers clear advantages in clinical application, particularly for patient groups with greater needs for drug safety and long-term prescription situations.

## Conclusion

In summary, this study demonstrates that SchC decreases lipid accumulation and enhances lipid metabolism, potentially due to its inhibition of the PI3K / AKT / mTOR autophagy pathway, which promotes autophagy and reduces pyroptosis in the liver tissue, ultimately reducing liver lipid accumulation. Additionally, SchC holds great promise as a potential alternative to traditional hypolipidemic drugs for the treatment of hyperlipidemia and associated pathological conditions. Nevertheless, further investigations are necessary to elucidate the precise molecular mechanism of how SchC functions as a hepatoprotective agent in clinical applications.

## Supplemental Materials

Supplementary data for this paper are available on-line only at http://jmb.or.kr.



## Figures and Tables

**Table 1:** Chronic stress procedures.

Stressors	Duration
Restraint stress (horizontal or vertical)	4 h
Removal of bedding	6 h
Wet bedding	6 h
Cage tilt 45°	6 h
Light overnight	12 h
Tail pinch	10 min

**Table 2:** List of antibodies.

Primary antibody	Catalog	Company
LC3A/B	#12741	CST
Beclin-1	#3495	CST
P62	#23214	CST
Phospho-mTOR (p-mTOR)	#2971	CST
mTOR	#4517	CST
Phospho-Akt (p-AKT)	#4060	CST
Phospho-PI3K (p-PI3K)	#17366	CST
Pro-Caspase-1	#24232	CST
Caspase-1	WL-03450	Wanleibio
NLRP3	#15101	CST
IL-1β	#12242	CST
IL-18	#57058	CST
Full length GSDMD (GSDMD)	WL 05411	Wanleibio
Cleaved N-terminal GSDMD (GSDMD-n)	WL 05411	Wanleibio
β-Actin	GB15003	Servicebio
Goat-anti-rabbit IgG	SA00001-2	Proteintech
