# Supplementary figures and images for: Schisandrin C Improves Chronic Stress-Induced Dyslipidemia in Mice by Regulating Pyroptosis and Autophagy Levels
Source: J Microbiol Biotechnol. 2025 Jun 23;35:e2501041. doi: 10.4014/jmb.2501.01041 (PMC12256839; doi:10.4014/jmb.2501.01041)

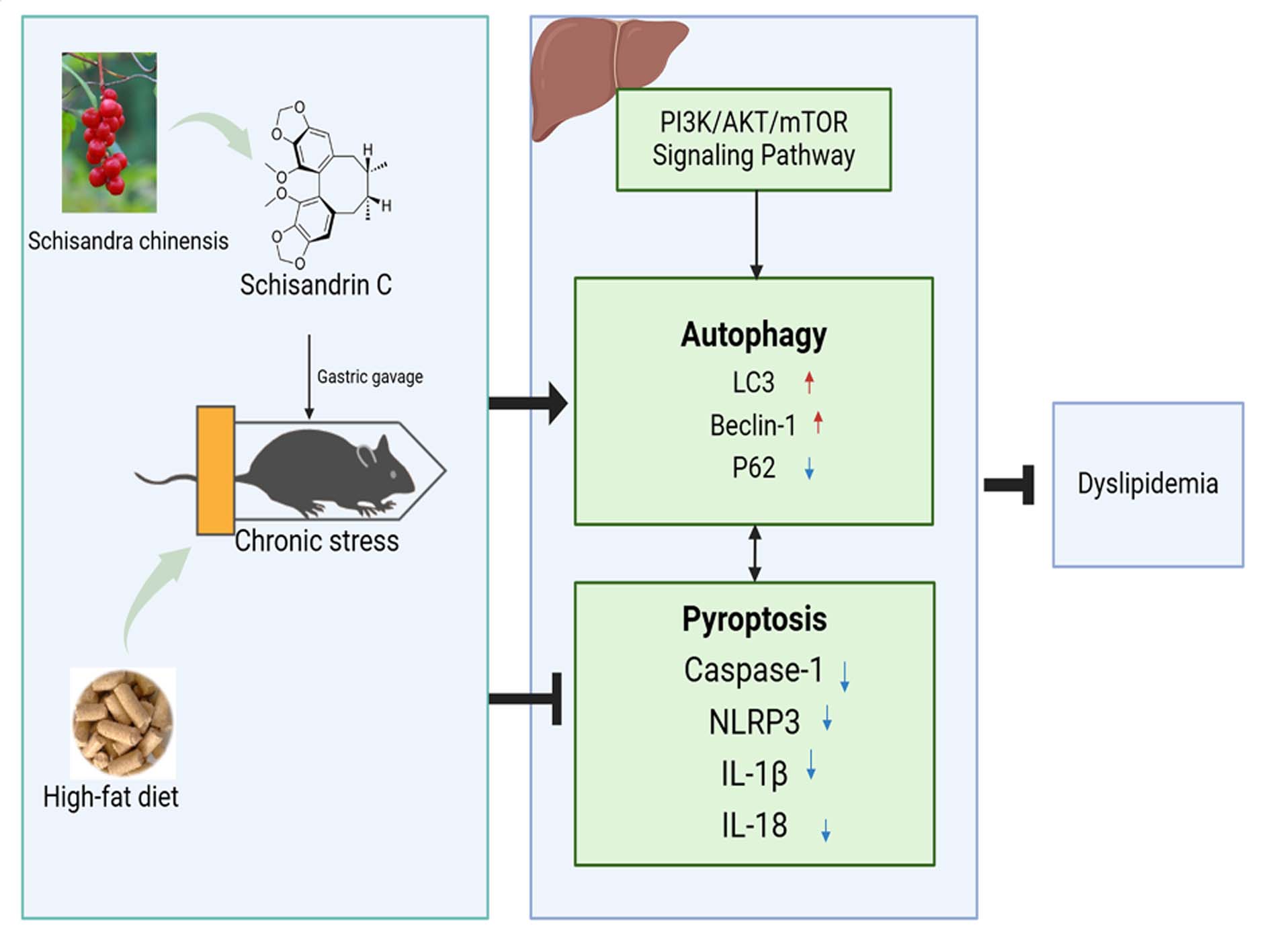

Supplement: Supplementary file 1 [file jmb-35-e2501041-supple.jpg]
